# Expression and Activity of Acid-Sensing Ion Channels in the Mouse Anterior Pituitary

**DOI:** 10.1371/journal.pone.0115310

**Published:** 2014-12-15

**Authors:** Jianyang Du, Leah R. Reznikov, Michael J. Welsh

**Affiliations:** 1 Howard Hughes Medical Institute, Roy J and Lucille A Carver College of Medicine, University of Iowa, Iowa City, Iowa, United States of America; 2 Department of Internal Medicine, Roy J and Lucille A Carver College of Medicine, University of Iowa, Iowa City, Iowa, United States of America; 3 Department of Molecular Physiology and Biophysics, Roy J and Lucille A Carver College of Medicine, University of Iowa, Iowa City, Iowa, United States of America; The Ohio State University, United States of America

## Abstract

Acid sensing ion channels (ASICs) are proton-gated cation channels that are expressed in the nervous system and play an important role in fear learning and memory. The function of ASICs in the pituitary, an endocrine gland that contributes to emotions, is unknown. We sought to investigate which ASIC subunits were present in the pituitary and found mRNA expression for all ASIC isoforms, including ASIC1a, ASIC1b, ASIC2a, ASIC2b, ASIC3 and ASIC4. We also observed acid-evoked ASIC-like currents in isolated anterior pituitary cells that were absent in mice lacking ASIC1a. The biophysical properties and the responses to PcTx1, amiloride, Ca^2+^ and Zn^2+^ suggested that ASIC currents were mediated predominantly by heteromultimeric channels that contained ASIC1a and ASIC2a or ASIC2b. ASIC currents were also sensitive to FMRFamide (Phe-Met-Arg-Phe amide), suggesting that FMRFamide-like compounds might endogenously regulate pituitary ASICs. To determine whether ASICs might regulate pituitary cell function, we applied low pH and found that it increased the intracellular Ca^2+^ concentration. These data suggest that ASIC channels are present and functionally active in anterior pituitary cells and may therefore influence their function.

## Introduction

Acid sensing ion channels (ASICs) are members of the epithelial sodium channel/degenerin superfamily that are activated by reduced extracellular pH. To date, six proteins of the ASIC family have been identified (ASIC1a, ASIC1b, ASIC2a, ASIC2b, ASIC3 and ASIC4). ASICs assemble as homo- or hetero-trimers to form proton-gated, voltage-insensitive, Na^+^ and Ca^2+^ permeable channels [1]–[9].

We recently reported that protons are a neurotransmitter and ASICs are a receptor that regulates synaptic transmission in the amygdala [10]. Disruption of the *ASIC1a* gene eliminates amygdala ASIC currents for pH values >5 and attenuates the acquisition of fear learning and memory. In contrast, overexpressing ASIC1a enhances fear conditioning (an animal model of acquired anxiety) [11]–[13]. These results suggested that ASICs might contribute to psychiatric disorders in which anxiety is a key component [11]. In addition, loss of ASIC currents in mice produces antidepressant-like effects [14].

While these data suggest that ASIC channels in the central nervous system might be involved in psychiatric illnesses, it is also possible that ASICs in other regions outside the CNS might also contribute. One such region is the anterior pituitary, an endocrine gland that secretes hormones critical for coordinating physiological responses to stress, as well as reproductive, metabolic, and developmental processes [15]. Indeed, an ASIC subunit, ASIC4, is highly expressed in the pituitary gland [16]. Although ASIC4 is not activated by a reduced pH, its presence suggests that other ASICs might play a role in pituitary function. In addition, similar to neurons, pituitary cells are excitable, express voltage-gated ion channels [17], [18], and release hormones in a Ca^2+^-dependent manner [18]–[20]. Thus, intracellular Ca^2+^, [Ca^2+^]_i_, in pituitary cells is crucial for overall pituitary function. ASICs are responsible for a proton-induced increase in [Ca^2+^]_i_ in many types of cells [21]–[23]. Therefore, ASICs were likely candidates to regulate pituitary gland function.

In this study, we asked whether ASICs are expressed in the pituitary gland and function as a proton receptor that detects changes in extracellular pH. Using a combination of reverse transcription polymerase chain reaction (RT-PCR), whole-cell patch-clamp recording, and [Ca^2+^]_i_ imaging, we identified ASIC expression and function in freshly isolated or cultured mouse anterior pituitary cells.

## Results

### RT-PCR analysis identified transcripts for ASIC subunits in mouse anterior pituitary cells

**Figure 1:**
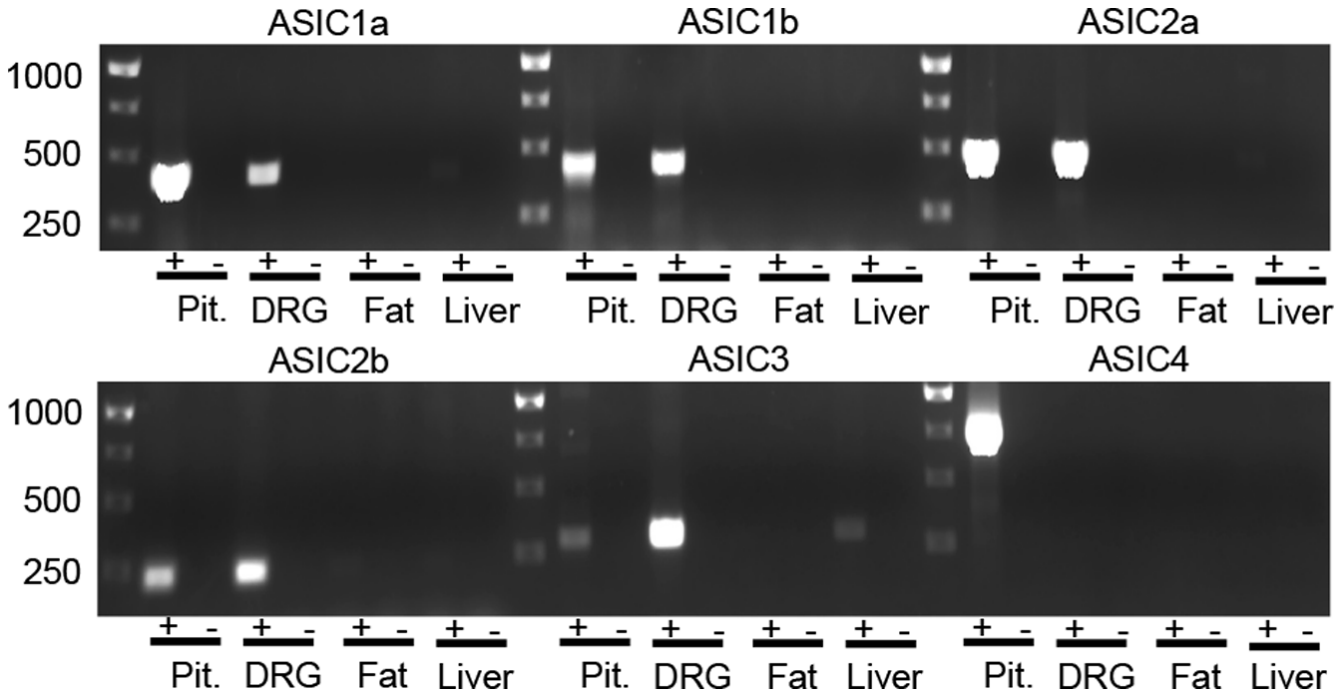
Detection of ASIC mRNA in freshly isolated mouse anterior pituitary tissue by reverse transcription polymerase chain reaction (RT-PCR). ASIC1a (product size 415b p), ASIC1b (product size 437 bp), ASIC2a (product size 481 bp), ASIC2b (product size 240 bp), ASIC3 (product size 300 bp), and ASIC4 (product size 740 bp) were all detected in cDNA of freshly isolated pituitary tissue. Positive and negative controls were performed using DRG, adipose and liver tissue.

To determine whether ASICs were present in the pituitary, we performed RT-PCR for ASIC subunits. We found mRNA for ASIC1a, ASIC1b, ASIC2a and ASIC2b in freshly isolated pituitary cells (Fig. 1). We also detected ASIC3 mRNA, although expression was low relative to dorsal root ganglion (DRG). We also detected expression of ASIC4, which is enriched in the pituitary compared to other organs [16].

### Acidic pH activated ASIC-like currents in freshly isolated mouse anterior pituitary cells

**Figure 2:**
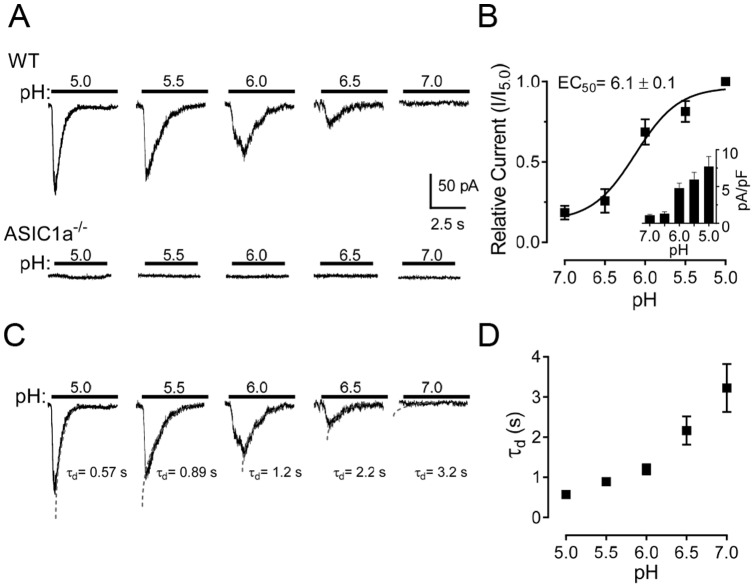
Acid-activated currents in freshly isolated mouse anterior pituitary cells. **A**. Representative ASIC currents elicited by solutions with the indicated pH in wild-type (WT) and *ASIC1a^-/-^* pituitary cells at −70 mV holding potential. The cells were held at pH 7.4 before and in between applications of acid. Note, no acid-activated currents were detected in *ASIC1a^-/-^* cells (n = 8 each). **B**. pH-dependent activation of ASIC currents. Best-fit yielded an EC_50_ pH of 6.1±0.1 (n = 8). Inset, the average current density of acid-activated currents. **C**. Example of pH-dependence of the decay time constant (τ_d_) of ASIC currents in anterior pituitary cells. The decay times were fitted by a single exponential equation. **D**. The pH-dependence of the decay time constant (τ_d_) (n = 5).

Because our PCR studies suggested that ASICs are expressed in the pituitary, we probed for ASIC function using patch-clamp electrophysiology. With a holding potential of −70 mV, we discovered that reducing extracellular pH from 7.0 to 5.0 activated a transient ASIC-like current in 83% (60 of 72 cells) freshly isolated mouse anterior pituitary cells (Fig. 2A). The amplitude of the transient current increased in a dose-dependent manner, with the average current density of acid-activated ASIC-like currents shown in the inset of Fig. 2B. Although the ASIC-like current amplitude was small compared to that in many neurons, the size of pituitary cells is also small (∼8 pF). Thus, the current density (pA/pF) is not far from that in neurons [10], [24]. In most pituitary cells, the threshold of pH to elicit ASIC-like current was 7.0, with the maximum response occurring at ∼5.0, although the current amplitude may not have completely saturated at pH 5 (Fig. 2A, 2B). The EC_50_ of pH was 6.1±0.1 (n = 8, Fig. 2B), which was slightly lower than the anticipated EC_50_ for homomeric ASIC1a (EC_50_  =  ∼6.2–6.8) or ASIC3 (transient component, EC_50_  =  ∼6.2–6.7) channels, but higher than the anticipated EC_50_ of homomeric ASIC2 (EC_50_  =  ∼4.1–5.0) channel [25]. Therefore, these results suggested that the ASIC-like current in pituitary cells is probably mediated via heteromultimeric channels, although these data do not exclude ASIC1b homomultimers (EC_50_  =  ∼5.1–6.2).

To confirm that the ASIC-like currents were conducted by ASIC channels, we applied a low pH (7.0 to 5.0) to pituitary cells from *ASIC1a^-/-^* mice and found that acid-activated currents were eliminated (Fig. 2A). These data suggest that the acid-induced current in anterior pituitary cells is conducted primarily through ASIC1a-containing channels and not ASIC3-containing channels as no ASIC-like currents persisted in *ASIC1a^-/-^* cells.

### Proton-gated currents had electrophysiological and pharmacological properties of ASICs

To further examine the biophysical properties of ASIC currents, we measured desensitization rates to solutions with varying pH values (Fig. 2C). The desensitization rate was measured by fitting the desensitization phase of the current with a single-exponential equation. Previous studies have shown that the decay times of ASICs decrease with lower pH [24], [26]–[28]. Similar to that earlier work, we found that the decay times of the ASIC currents were pH-dependent (Fig. 2D). The decay time constant (τ_d_) for ASIC currents activated at pH 5.0 was 0.57±0.04 s, at pH 5.5 was 0.89±0.06 s, at pH 6.0 was 1.20±0.13 s, at pH 6.5 was 2.20±0.35 s, and at pH 7.0 was 3.22±0.60 s (n = 5, Fig. 2C, 2D).

**Figure 3:**
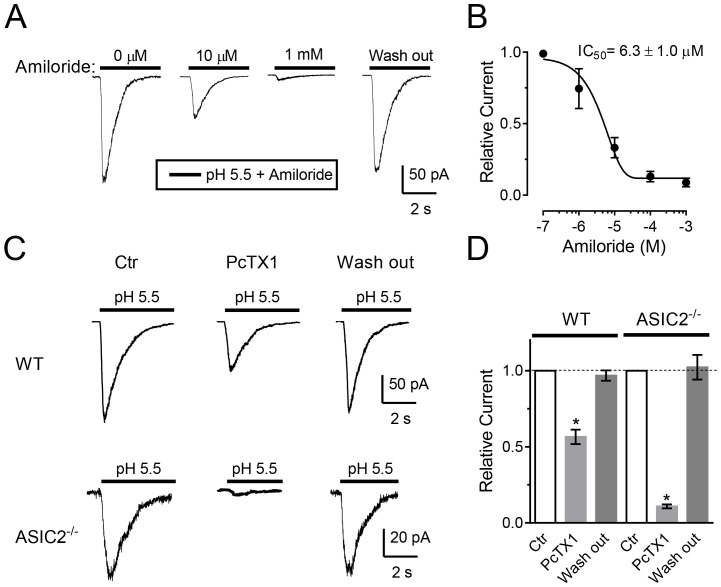
The effects of ASIC channel blockers on ASIC currents in freshly isolated anterior pituitary cells. **A**. Representative ASIC currents elicited by pH 5.5 with various concentrations of amiloride. Holding potential was −70 mV. The cells were held at pH 7.4 before and in between applications of pH 5.5. Amiloride was applied during acid application. **B**. Summary data showing the dose-dependent block of ASIC currents by amiloride in anterior pituitary cells (n = 7). Currents were normalized to the control condition (without amiloride). The IC_50_ of amiloride block was 6.3±1.0 µM (n = 7). C. Representative ASIC currents in WT and ASIC2^-/-^ cells elicited by pH 5.5 with 100 nM PcTx1. Cells were pre-treated with PcTx1 in pH 7.4 for 1 min and then were perfused with pH 5.5 containing 100 nM PcTx1. Holding potential was −70 mV. D. Summary data showing the block of ASIC currents by 100 nM PcTx1 in anterior pituitary cells from WT (n = 7) and ASIC2^-/-^ mice (n = 6). * *P*<0.05 compared to control, Student's *t*-test.

Amiloride, a non-specific ASIC blocker, inhibits ASIC currents in many cell types [1], [24], [26], [27], [29]–[33]. We found that amiloride also inhibited ASIC currents in anterior pituitary cells (Fig. 3). Amiloride's effects were dose-dependent (Fig. 3A) with an IC_50_ of 6.3±1.0 µM (Fig. 3B). This sensitivity is slightly higher than that for recombinant homomeric ASIC channels (ASIC1a ∼10 µM; ASIC1b ∼21–23 µM; ASIC2a ∼28 µM; ASIC3 ∼16–63 µM) [25]. The amiloride-sensitivity we observed in anterior pituitary cells is consistent with previous studies showing variable blockage by amiloride of ASICs in HEK 293 cells (IC_50_ of 2.2 µM) [32], rat suprachiasmatic nucleus neurons (IC_50_ of 14 µM) [33], PC12 cells (IC_50_ of 0.68 µM) [26], and dorsal horn neurons (IC5_0_ of 16.2 µM) [30].

To further identify the ASIC subunits, we applied PcTx1, which blocks homomeric ASIC1a and heteromeric ASIC1a/2b channels [34], [35]. PcTx1 (100 nM applied 1 min before a pH reduction) inhibited the response to pH 5.5 by 44±5% (Fig. 3C, 3D). We also tested the effect of PcTx1 on ASIC2^-/-^ anterior pituitary cells and found that 100 nM PcTx1 inhibited 90±2% of pH 5.5-induced current (Fig. 3C, 3D). PcTx1 inhibition of about half the current suggests that either homomeric ASIC1a or ASIC1a/2b channels contribute to the H^+^-evoked current because PcTx1 inhibits those channels [34], [35]. Finding that about half the current was PcTx1-insensitive suggests that ASIC1a/2a channels contribute. Because PcTx1 blocked most of the current in ASIC2^-/-^ cells, ASIC1a/1b and ASIC3 likely make little, if any, contribution to the current because those channels are PcTx1-insensitive.

**Figure 4:**
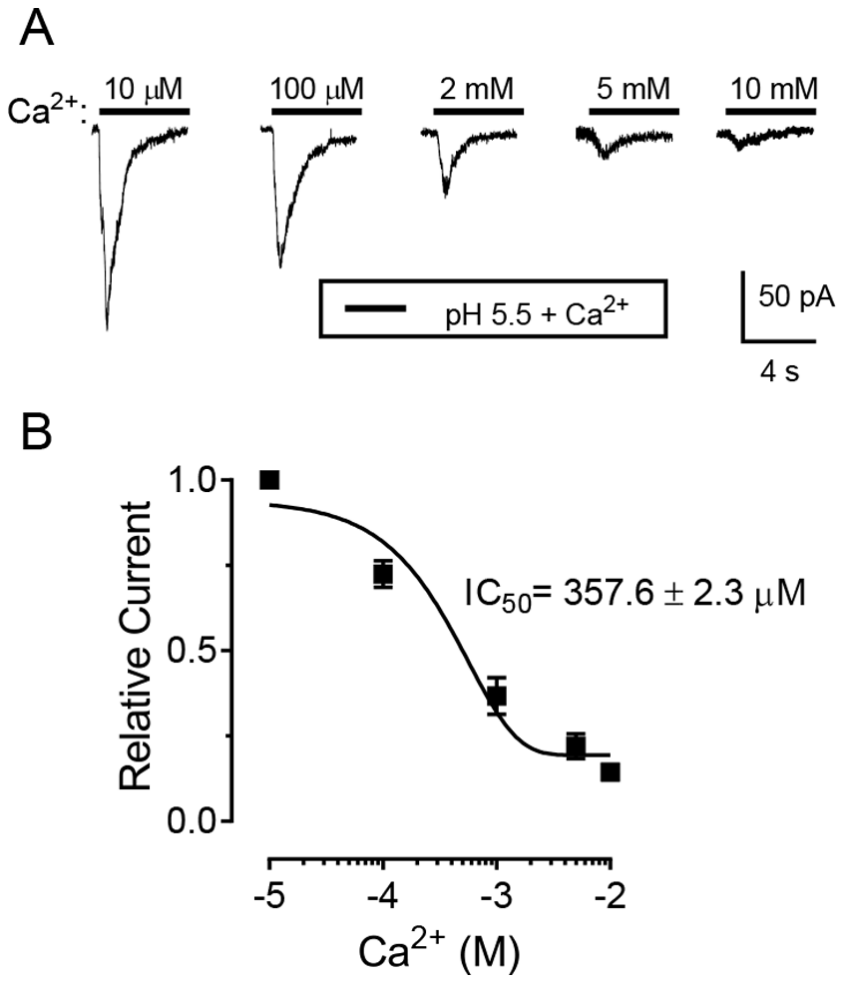
The effects of extracellular Ca^2+^ on ASIC currents in freshly isolated anterior pituitary cells. **A**. Representative ASIC currents elicited by pH 5.5 with various concentrations of extracellular Ca^2+^. Holding potential was −70 mV. Ca^2+^ concentrations from 0 µM to 10 mM at pH 7.4 were applied for 2 minutes and ASIC currents were activated by pH 5.5 for 5 sec with the same Ca^2+^ concentration. **B**. Summary data showing the dose-dependent block of ASIC currents by Ca^2+^. The IC_50_ of Ca^2+^ block was 357±20 µM (n = 7).

ASICs have also been shown to exhibit sensitivity to extracellular Ca^2+^ [36]. The increase in extracellular Ca^2+^ inhibits ASIC1a and ASIC3 currents by shifting the pH-dependence of activation to more acidic values. An elegant model also suggested that Ca^2+^ blocks the open pore of ASIC [36], [37]. We tested the effects of varying the extracellular Ca^2+^ concentration on ASIC currents in anterior pituitary cells. We perfused with a bath solution that contained 2 mM Ca^2+^ and evoked ASIC currents with a pH of 5.5. Increasing the extracellular Ca^2+^ concentration reduced ASIC currents, whereas decreasing Ca^2+^ enhanced ASIC currents (Fig. 4A). The best-fit analysis revealed an IC_50_ of 357±20 µM (Fig. 4B). This value is much lower than that reported in several previous studies (dorsal horn neurons, IC_50_ is 4.1 mM [30]; hippocampus CA1 neurons, IC_50_ is ∼2–5 mM [38]; recombinant ASIC1a, IC_50_ is 3.9±1.0 mM [37]. Thus, we suspect that pituitary ASICs might have unique heteromultimeric channel and/or associated protein combinations that contribute to its Ca^2+^ sensitivity.

**Figure 5:**
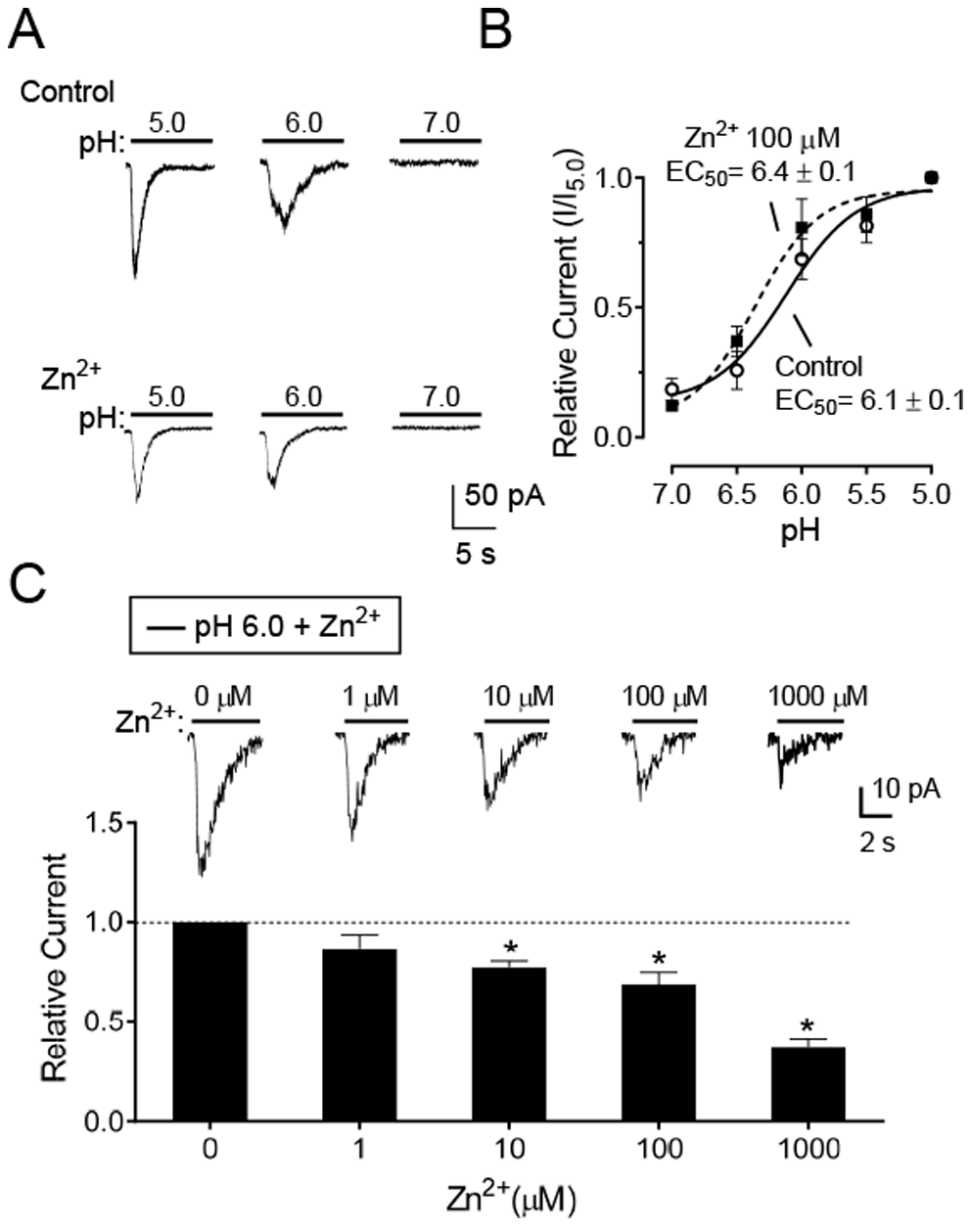
The effects of extracellular Zn^2+^ on ASIC currents in freshly isolated anterior pituitary cells. **A.** Representative pH-dependent activation of ASIC currents in control without (control, upper) and with (lower) 100 µM Zn^2+^. Cells were perfused with 100 µM Zn^2+^ at pH 7.4 for 2 min, and then were tested with solutions at the indicated pHs (∼5 sec) that also contained 100 µM Zn^2+^. **B.** Best-fit of the pH-dependent activation of ASIC currents yielded an EC_50_ pH of 6.1±0.1 (control, n = 8) and 6.4±0.1 (with 100 µM Zn^2+^, n = 8). **C.** Top, representative data of the dose-dependent effect of Zn^2+^ on ASIC current amplitude. Zn^2+^ concentrations from 0 µM to 1 mM at pH 7.4 were applied for 2 minutes and ASIC currents were activated by 5 s of pH 6.0 with the same Zn^2+^ concentration. Bottom, average data of the inhibitory effects of Zn^2+^ (n = 9). * *P*<0.05, compared with 0 µM Zn^2+^ group, one-way ANOVA with Dunnett's multiple comparison test.

Previous studies indicate that Zn^2+^ potentiates homomeric and heteromultimeric channels that contain ASIC2a (ASIC2a, ASIC1a/2a, and ASIC2a/3) in the micromolar range [27], [39], but inhibit homomeric ASIC1a channels in the nanomolar range and ASIC3 channels in the micromolar range [40], [41]. Thus, to gain further insight into pituitary ASIC channel properties, we tested the effects of Zn^2+^ on freshly isolated anterior pituitary cells. Similar to previous studies in heteromeric ASIC1a/2a channels [39], we found that 100 µM Zn^2+^ increased the pH-sensitivity of ASIC current with a shift in the pH EC_50_ from 6.1±0.1 (control) to 6.4±0.1 (with 100 µM Zn^2+^) (Fig. 5A, 5B). However, in contrast to studies of recombinant ASIC1a/2a subunits expressed in oocytes, micromolar Zn^2+^ reduced the peak amplitude of ASIC currents (Fig. 5C). Thus, while the increased pH-sensitivity is consistent with data from recombinant ASIC1a/2a heteromultimers, the decreased amplitude is not. The reason for the difference is unknown, but suggests that the endogenous system may be more complex than recombinant system. Interestingly, micromolar Zn^2+^ has been reported to reduce current amplitude in the rat suprachiasmatic nucleus [33].

### FMRFamide modulated ASIC currents in anterior pituitary cells

**Figure 6:**
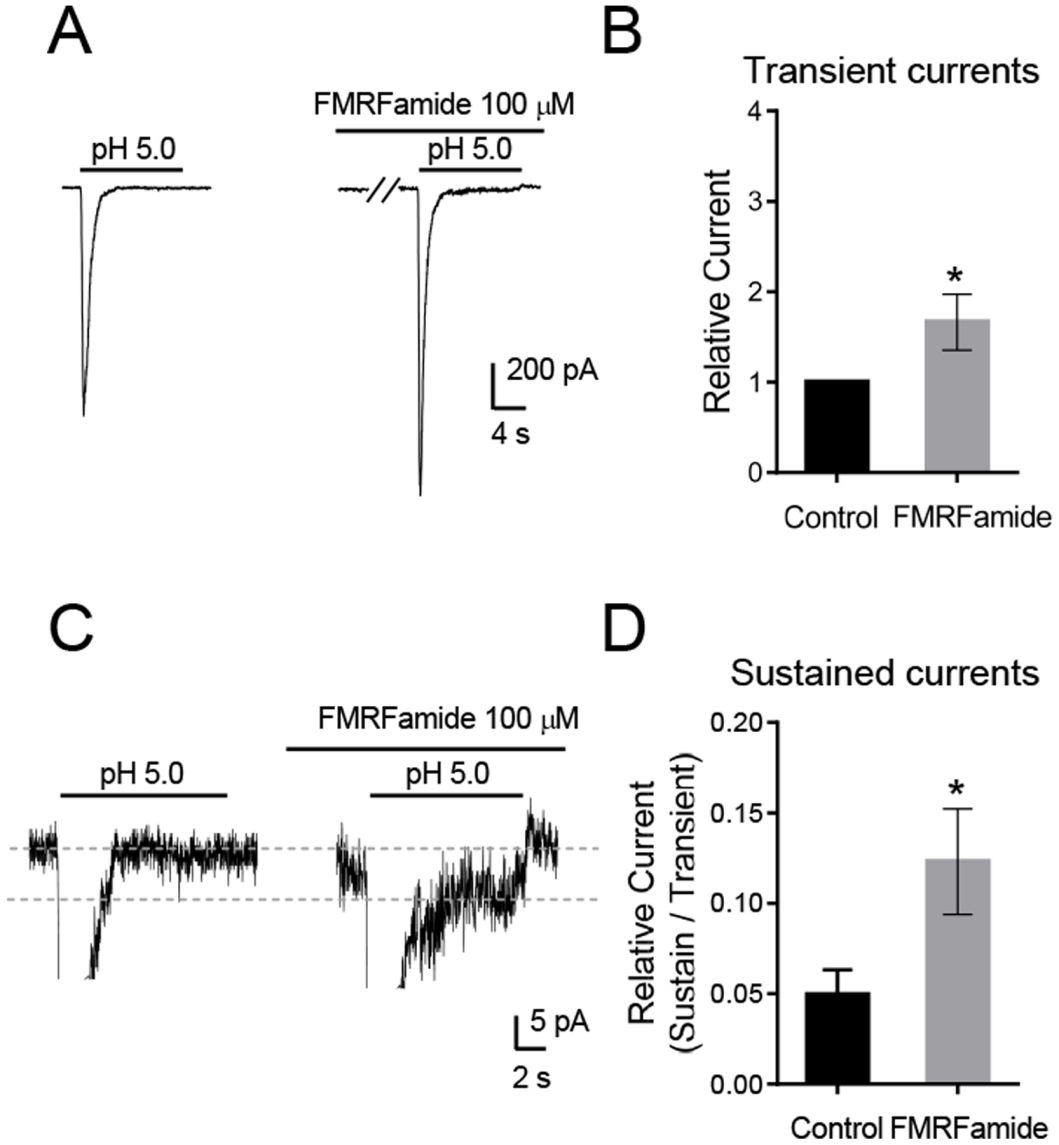
Effects of FMRFamide on ASIC currents in freshly isolated anterior pituitary cells. **A, C.** Representative traces of pH 5.0 activated ASIC currents, with and without 30 second pretreatment with 100 µM FMRFamide. FMRFamide potentiated the pH 5.0 induced peak (**A**) and sustained (**C**) currents. **B, D.** Average data showing potentiation of current by FMRFamide as shown in **A** and **C** (n = 7). * *P*<0.05, Student's *t*-test.

FMRFamide (Phe-Met-Arg-Phe amide) is a neuropeptide that acts as neurotransmitter and neuromodulator in invertebrates. Askwith et al reported that pretreatment with 100 µM FMRFamide potentiated ASIC currents [42]. To determine whether ASICs in anterior pituitary cells also display this property, we pretreated cells with 100 µM FMRFamide for 30 sec and found significant potentiation of the transient component of ASIC current (66±31% more than control, n = 7) (Fig. 6A, 6B). In addition, the sustained component of the ASIC current is increased after FMRFamide (Fig. 6C, 6D). These results suggest that pituitary ASICs might be modulated by endogenous FMRFamide-like neurotransmitters.

### Application of acid to anterior pituitary cells induced a [Ca^2+^]_i_ increase

**Figure 7:**
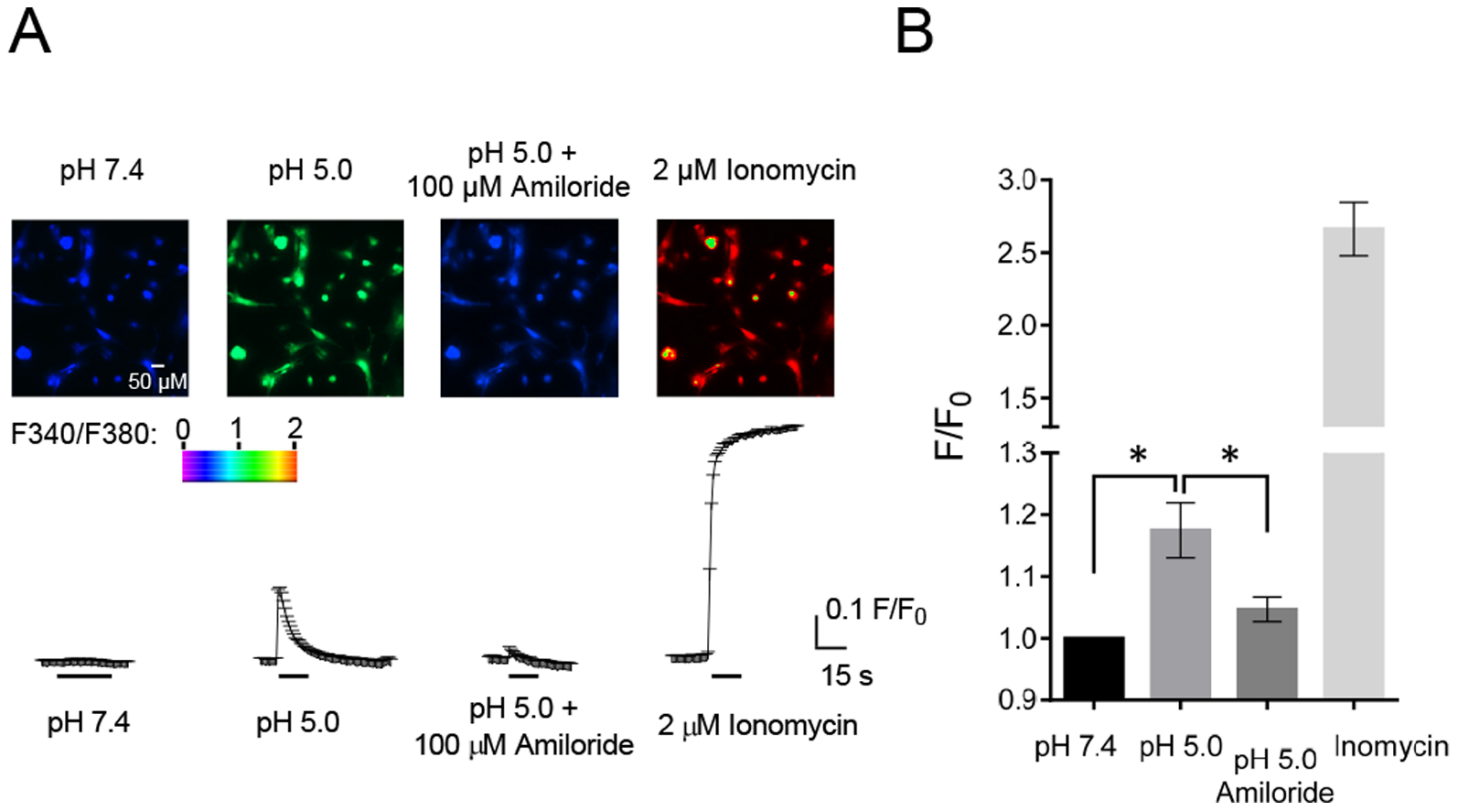
Acid-induced [Ca^2+^]_i_ increase in cultured anterior pituitary cells. **A.** Fluorescence (F340/F380 ratio) was measured under indicated conditions. Top, representative image under indicated conditions. Bottom, average traces of F/F_0_, (F340/F380)/(F340/F380 at pH 7.4). Applications of 2 µM ionomycin at the end of the recordings served as a positive control (n = 6). **B**. Average values of F/F_0_. pH 5.0 increased the F340/F380 ratio to 118±5% compared to the ratio at pH 7.4. Amiloride inhibited the pH 5.0 induced ratio changes (104±2% as baseline), n = 6. * *P*<0.05, one-way ANOVA with Tukey's post-hoc multiple comparison test.

An increased [Ca^2+^]_i_ controls hormone release in anterior pituitary cells [18], [43]. Because activation of ASIC1a-containing heteromultimeric channels can increase [Ca^2+^]_i_ [21]–[23], we hypothesized that extracellular application of acid in anterior pituitary cells might increase [Ca^2+^]_i_. We used a ratiometric Ca^2+^ imaging method to detect changes in [Ca^2+^]_i_. Decreasing pH from 7.4 to 5.0 triggered a transient increase in [Ca^2+^]_i_ in 82% (124/152 cells) of cultured mouse anterior pituitary cells (Fig. 7). Consistent with activation of ASICs, the increase in [Ca^2+^]_i_ was inhibited by 100 µM amiloride. Previous studies indicate that activation of ASICs might increase [Ca^2+^]_i_ either directly by allowing Ca^2+^ influx [21], [23], [44] or indirectly by depolarizing the cell membrane and activating voltage-gated Ca^2+^ channels [22]. Our data do not discriminate between these two mechanisms.

## Discussion

Our RT-PCR analysis of pituitary identified transcripts for six ASIC subunits: ASIC1a, ASIC1b, ASIC2a, ASIC2b, ASIC3 and ASIC4. The electrophysiological experiments then established that anterior pituitary cells expressed ASIC currents. The following biophysical and pharmacological properties of the acid-induced currents suggested which ASIC subunits might contribute to the H^+^-gated currents.

First, because disrupting the *ASIC1a* gene eliminated the currents, we conclude that ASIC1 subunits are a key component of the H^+^-gated currents, and ASIC3 made little if any contribution. If ASIC3 were involved, then acid-evoked currents should have persisted in *ASIC1a^-/-^* anterior pituitary cells. In addition, ASIC3 transcripts were reduced in anterior pituitary compared to dorsal root ganglia.

Second, results from PcTx1 experiments have implications for the subunits involved. a) Homomeric ASIC1a and/or ASIC1a/2b channels might contribute to the current because PcTx1 inhibited about half the current, and PcTx1 inhibits those channels [34], [45]. b) ASIC1a/2a channels are likely to contribute, because PcTx1 inhibited only about half the current, and ASIC1a/2a subunits are PcTx1-insensitive. c) ASIC1b, ASIC1a/1b and ASIC3 likely make little, if any, contribution to the current in anterior pituitary cells because PcTx1 blocked most of the current in ASIC2^-/-^ cells and those channels are PcTx1-insensitive.

Third, the properties of the H^+^-evoked currents suggested that neither ASIC1 nor ASIC2 homomultimers generated the acid-activated current. For example, the EC_50_ for pH activation of anterior pituitary H^+^-gated currents (pH 6.1±0.1) was much higher than that for ASIC2a homomultimers (pH 4.1–5.0) [3], [8], [28], [46] and ASIC2b homomultimers do not generate current [3], [6], [8]. Although the EC_50_ for pH activation of anterior pituitary cell currents was in the range observed with ASIC1a (pH 5.8–6.8) [1], [34], [46]–[52] and ASIC1b (pH 5.1–6.2) [46], [47], [53] homomultimers, the current decay time we observed (0.57 sec) is slower than that for ASIC1a (0.25 sec) or ASIC1b (0.44 sec) homomultimers [28].

Fourth, the IC_50_ for amiloride inhibition of ASIC currents in anterior pituitary cells (6.3 µM) was lower than for ASIC1a or ASIC1b homomultimers (10 µM and 21–23 µM, respectively) [25].

Fifth, the EC50 of pH activation of anterior pituitary cell currents (pH 6.1) is in the range of that for homomeric ASIC1a (EC_50_  =  pH 5.8) and ASIC1a/2a (EC_50_  =  pH 5.5) and ASIC1a/2b (EC_50_  =  pH 6.2) heteromultimers [28]. Currents generated by ASIC heteromultimers would be consistent with previous studies indicating that heteromultimers of subunits account for most neuronal H^+^-gated current in DRG neurons [46], [54], hippocampal neurons [31], [35], [55], cerebellar purkinje neurons [29], suprachiasmatic nucleus neurons [33], PC12 cells [26], cortical neurons [24], olfactory bulb mitral/tufted neurons [27] and dorsal horn neurons [30].

Sixth, the effects of Ca^2+^ and Zn^2+^ would not be predicted for ASIC1 homomultimers. Extracellular Ca^2+^ inhibited H^+^-gated current with an IC_50_ of 357±20 µM, lower than that reported for ASIC1a (3.9±1.0 mM) or ASIC1b (>10 mM) homomultimers [37]. In addition, Zn^2+^ shifted the pH EC_50_ of anterior pituitary ASIC currents to more alkaline values; previous work showed that Zn^2+^ increased the pH-sensitivity of heteromultimeric ASIC1a/2a channels in the micromolar range [39]. Micromolar Zn^2+^ also reduced ASIC current amplitude; the explanation is uncertain, but Zn^2+^ can inhibit ASIC3 in the micromolar range [40], [41].

Taken together, the data are most consistent with H^+^-gated currents generated by ASIC1a/2a and ASIC1a/2b heteromultimers and ASIC1a homomultimers. However, not every property of the acid-evoked currents aligned precisely with the properties observed in studies of recombinant ASIC subunits expressed in heterologous cells. Thus, some uncertainty exists when comparing data from recombinant and endogenous ASIC currents.

ASIC currents in anterior pituitary cells were unexpectedly sensitive to extracellular Ca^2+^ compared to results obtained with cells from other brain regions. For example, previous studies in dorsal horn and CA1 hippocampal neurons suggest that extracellular Ca^2+^ inhibits proton-gated currents in a concentration-dependent manner with an IC_50_ of 4.1 mM [30] and 2–5 mM, respectively [38]. This contrasts with the IC_50_ of Ca^2+^ (357±2 µM) on pituitary cell ASICs. We suspect that the ASIC subunits or associated proteins in pituitary cells might have some differences from those in other neurons. One possibility is that this behavior is conferred by ASIC4, which is highly expressed in the pituitary gland. Arguing against that possibility is the lack of evidence to support a function of ASIC4 in regulating other ASIC subunits.

We recently reported that protons are a neurotransmitter and ASICs are a postsynaptic receptor in amygdala neurons [10]. Our current data suggest that this ligand-receptor pair may also serve an important role in the function of anterior pituitary cells. Finding a proton-induced increase in [Ca^2+^]_i_ is suggestive, because Ca^2+^ is a crucial trigger for hormone secretion in pituitary cells [18], [20]. Pituitary cells store peptide hormones in vesicles and an increased Ca^2+^ initiates their exocytosis into the extracellular space. It is also interesting that FMRFamide, a neurotransmitter and neuromodulator in invertebrates, regulates anterior pituitary cell ASIC currents. Although FMRFamide is not present in vertebrates, other RFamides have been reported in the vertebrate central nervous system and the endogenous RFamide-related peptides inhibit ASIC1a steady-state desensitization [45], [56], [57]. Thus, FMRFamide-like peptide modulation of ASICs might also influence anterior pituitary function.

Discovering ASIC channel currents in anterior pituitary cells suggests that they may influence pituitary cell hormone secretion. However, it remains uncertain whether or not the anterior pituitary has physiological variations in extracellular pH. We speculate that variations in local metabolic activity may transiently reduce pH in a manner similar to what can occur in the brain [21], [58], [59]. If so protons and ASICs may regulate anterior pituitary function. This might be especially important for depression and anxiety, in which an altered hypothalamic-pituitary-adrenal axis (HPA axis) has been implicated [60], [61].

## Methods

### Mice and ethic statement

*ASIC1a^-/-^* mice were described previously [62]. *ASIC1a^-/-^* and wild-type mice were maintained on a congenic C57BL/6 background. Experimental groups were matched for age (8–10 weeks old). Mice were kept on a standard 12 hour light-dark cycle and received standard chow (LM-485; Teklab, Madison, WI, USA) and water ad libitum. The University of Iowa Animal Care and Use Committee approved all procedures. All efforts were made to minimize suffering.

### Anterior pituitary cell isolation and culture

Mice were anesthetized with isoflurane, the pituitaries were removed, they were washed three times with DMEM/F12 medium (Invitrogen), and the posterior lobes were removed under a dissection microscope. The anterior lobes were then incubated with stirring in a hank's balanced salt solution (HBSS) containing 0.5% collagenase type 1 (Sigma-Aldrich) and 1% bovine serum albumin (BSA) at 37°C for 10 to 25 min. The tissues were then switched to a new HBSS containing 0.25% trypsin (Life Technologies) and digested at 37°C for 5 min. After digestion, cells were placed in fresh DMEM/F12 medium containing 10% fetal bovine serum (FBS) and pipetted up and down with P-1000 until much of the tissue was broken up. Some of the isolated cells were then seeded onto coverslips for 1 hour for use in electrophysiological recordings. The remaining cells were seeded into 35 mm culture dishes and cultured for 1–2 weeks at 37°C in a humidified 5% CO_2_ atmosphere incubator. The cultured anterior pituitary cells were used for Ca^2+^ imaging.

### Patch-clamp recording

Freshly isolated anterior pituitary cells were used for patch-clamp recordings. The pipette solution for whole-cell patch clamp recordings contained (in mM) 135 Cs-methanesulfonate (CsSO_3_CH_3_), 8 NaCl, 1 EGTA, and 10 HEPES (mOsm∼290, adjusted to pH 7.25 with CsOH). The standard extracellular bath solution for whole-cell recording contained (mM): 145 NaCl, 5 KCl, 2 CaCl_2_, 10 HEPES and 10 glucose, pH was adjusted to 7.4 with NaOH. In some experiments, 5 mM EGTA was added to the bath solution to buffer Ca^2+^ concentration. MaxChelator software (http://www.stanford.edu/~cpatton/webmaxcS.htm) was used to calculate free Ca^2+^ concentrations. The acidic pH solutions were prepared as described previously [10]. In brief, the 10 mM HEPES that was used in the solutions at pH 7.4, 7.0 and 6.5 was replaced by 10 mM MES for the solutions at pH≤6. All chemicals were ordered from Sigma-Aldrich. Psalmotoxin-1 (PcTX1) was ordered from Peptides International. In most cases, we analyzed the current amplitude, not current density. For the analysis of current amplitude, we did not analyze cells with “run down” of greater than 20% of the original amplitude.

### Ratiometric Ca^2+^ imaging

We used cultured cells for [Ca^2+^]_i_ imaging because they adhere to the coverslip much better than acutely isolated cells. The cultured anterior pituitary cells on glass coverslips were loaded with 5 µM of the ratiometric Ca^2+^ dye Fura-2 +0.02% Pluronic-F127 at room temperature for 1–1.5 hour. Fura-2 has an emission peak at 505 nm and dual excitation peaks at 340 nm and 380 nm. The 340/380 nm excitation ratio of fura-2 reports changes in the intracellular Ca^2+^ concentration. Cells were then washed with the bath solution containing (in mM) 140 NaCl, 4.7 KCl, 1.2 MgCl_2_, 1.2 KH_2_PO_4_, 1.3 CaCl_2_, 10 HEPES, 10 glucose, and pH was adjusted to 7.35 with NaOH. [Ca^2+^]_i_ was measured by perfusing the cells with the patch-clamp bath solution (pH 7.4 and pH 5.0) described above. At the end of each measurement, 2 µM ionomycin was applied as an internal control. The fluorescence of 340∶380 nm ratio was measured and data were analyzed using NIS-Elements (Nikon).

### RT-PCR analysis

Total RNA was isolated from freshly dissected anterior pituitary tissue using Qiagen RNAeasy kits and reverse transcribed using VILO mastermix. Primer sequences for amplifying ASIC1a, 1b, 2a, 2b, 3 and ASIC4 subunits were previously described [63] [64]. Primers were used for growth hormone were: FWD: 5′CAGCCTGATGTTTGGTACCTCGGA3′; RVS: 5′GCGGCGACACTTCATGACCCGCA3′. 25ng of cDNA was used and PCR amplification was performed according to the manufacturer's instructions. Products were run on an agarose gel and visualized with UV light.

### Data Analysis

Data are presented as means ± SEM. Dose-response curves were fitted by Prism software with an equation of *E = E_max_ {1/[1+(EC_50_/C)^n^]}*, where E is the effect at concentration C, Emax is maximal effect, EC_50_ is the concentration for half-maximal effect, and n is the Hill coefficient. The Hill coefficient value was not constrained in the analyses. Statistical comparison of groups used one-way ANOVA and Tukey's post-hoc multiple comparison test. A Student's t-test was used to compare two groups. P<0.05 indicated statistical significance.
